# Effect of Porosity and Heat Treatment on Mechanical Properties of Additive Manufactured CoCrMo Alloys

**DOI:** 10.3390/ma16020751

**Published:** 2023-01-12

**Authors:** Tu-Ngoc Lam, Kuang-Ming Chen, Cheng-Hao Tsai, Pei-I Tsai, Meng-Huang Wu, Ching-Chi Hsu, Jayant Jain, E-Wen Huang

**Affiliations:** 1 Department of Materials Science and Engineering, National Yang Ming Chiao Tung University, Hsinchu 30010, Taiwan; 2 Department of Physics, College of Education, Can Tho University, Can Tho City 900000, Vietnam; 3 Biomedical Technology and Device Research Laboratories, Industrial Technology Research Institute, Chutung, Hsinchu 310401, Taiwan; 4 Department of Orthopaedics, Taipei Medical University Hospital, Taipei 11031, Taiwan; 5 Department of Orthopedics, College of Medicine, Taipei Medical University, No. 250, Wuxing St., Xinyi District, Taipei 11031, Taiwan; 6 Department of Mechanical Engineering, National Taiwan University of Science and Technology, Taipei 106, Taiwan; 7 Department of Materials Science and Engineering, Indian Institute of Technology, New Delhi 110016, India

**Keywords:** selective laser melting, CoCrMo, porosity, heat treatment, compression test

## Abstract

To minimize the stress shielding effect of metallic biomaterials in mimicking bone, the body-centered cubic (bcc) unit cell-based porous CoCrMo alloys with different, designed volume porosities of 20, 40, 60, and 80% were produced via a selective laser melting (SLM) process. A heat treatment process consisting of solution annealing and aging was applied to increase the volume fraction of an ε-hexagonal close-packed (hcp) structure for better mechanical response and stability. In the present study, we investigated the impact of different, designed volume porosities on the compressive mechanical properties in as-built and heat-treated CoCrMo alloys. The elastic modulus and yield strength in both conditions were dramatically decreased with increasing designed volume porosity. The elastic modulus and yield strength of the CoCrMo alloys with a designed volume porosity of 80% exhibited the closest match to those of bone tissue. Different strengthening mechanisms were quantified to determine their contributing roles to the measured yield strength in both conditions. The experimental results of the relative elastic modulus and yield strength were compared to the analytical and simulation modeling analyses. The Gibson–Ashby theoretical model was established to predict the deformation behaviors of the lattice CoCrMo structures.

## 1. Introduction

Metallic materials, such as tantalum (Ta)-based, titanium (Ti)-based, and cobalt (Co)-based alloys, are widely used as bone implants. Among the most prevalent materials used for promising biomedical applications [1], cobalt-chromium-molybdenum (CoCrMo) alloys [2] have attracted great interest due to their superior biocompatibility, corrosion resistance, wear resistance, and good mechanical properties [3,4,5,6,7,8,9,10]. Poor tribological behavior caused by a high friction coefficient and wear debris is one of the crucial obstacles for Ti-based alloys [11], which can be overcome by the use of CoCrMo alloys. Extensive research on the mechanical and microstructural properties of widely used cast, wrought, or hot-forged CoCrMo alloys has been reported [6,12,13,14,15]. To fulfill the criteria for metallic materials as feasible implants, it is important to reduce the stress shielding effect and enhance osseointegration [16,17,18,19,20,21] by applying porous lattice structures, which are difficult to achieve via traditional fabrication processes.

The wear resistance of CoCrMo alloys is governed by the amount of carbon, the homogeneous distribution of carbides, and the existence of a hexagonal close-packed (hcp) structure [22]. There may exist two different crystal structures—metastable γ-face-centered cubic (fcc) and ε-hcp—in the CoCrMo alloys at room temperature, and their volume fraction can be changed via heat treatment conditions [23,24,25]. Increasing the volume fraction of the hcp phase is beneficial to the improved mechanical and wear properties of CoCrMo alloys, as well as to their stability [24,25,26,27,28]. A number of studies on various process- and heat-treatment conditions have been devoted to promoting a martensitic transformation from fcc to hcp [23,24,25,26,29,30].

Additive manufacturing allows for complex structures produced with diverse geometries and shapes associated with controllable microstructures [31,32,33,34,35]. Better metallurgical design can be achieved through machine learning and high-throughput examinations [36]. Among the most common three-dimensional (3D) printing processes, selective laser melting (SLM) produces SLM-built metallic parts with distinct microstructures [37,38,39]. In addition, SLM offers great possibilities in tailoring porous lattice structures with various unit cell types, cell sizes, and strut dimensions, which enables the tuning of the mechanical properties of SLM-built metallic implants to closely match those of human bone [39,40]. Comprehensive research has been conducted on the mechanical properties of additive-manufactured porous CoCrMo alloys via different, designed volume porosities [41,42,43] or heat treatment conditions to reduce the stiffness mismatch between bone and biomedical CoCrMo implants [24,25,44]. Furthermore, the analytical and simulation modeling analyses were generally used to predict the mechanical properties of porous structures [18,45,46]. However, investigating the ideal porosity and pore sizes for porous implants is still controversial [47], and exploring the role of different, designed volume porosities on heat-treated CoCrMo alloys fabricated via SLM is limited.

In the present study, the designed lattice structure of body-centered cubic (bcc) unit cell-based porous CoCrMo alloys was manufactured via SLM. The objective of this work was to discover the optimal design parameters for a closer match between bone tissue and CoCrMo alloys. The influence of different, designed volume porosities on the compressive mechanical properties of as-built and heat-treated CoCrMo alloys was examined. Moreover, the theoretical model proposed by Gibson–Ashby was employed to predict the mechanical behavior of porous SLM-built CoCrMo structures, which is conducive to establishing the optimal design of bcc lattice structures with suitable mechanical properties for biomedical applications.

## 2. Materials and Methods

### 2.1. Sample Preparation

**Figure 1:**
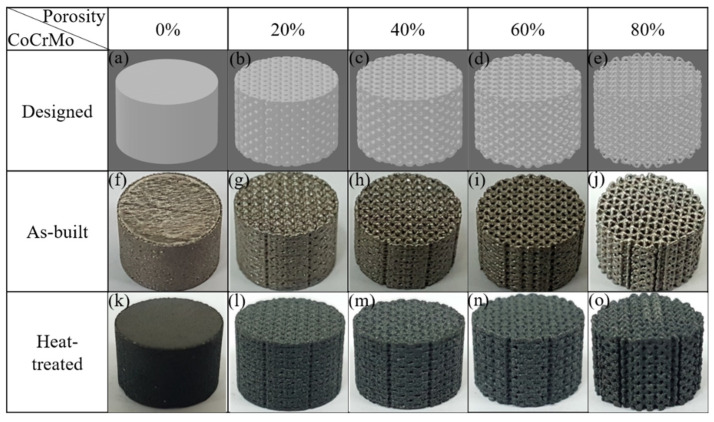
(**a**–**e**) CAD-designed models, (**f**–**j**) as-built, and (**k**–**o**) heat-treated CoCrMo alloys with different, designed volume porosities.

The cylindrical shapes of as-built porous CoCrMo alloys with a diameter of 11 mm and a height of 7 mm were fabricated using the SLM AM100 machine with a working space of 10 cm × 10 cm manufactured by the Industrial Technology Research Institute (ITRI). The diameters of the struts were 0.2, 0.3, 0.4, and 0.5 mm, corresponding to the designed volume porosity of 80, 60, 40, and 20%, respectively. Figure 1a–e shows computer aided design (CAD) models for the designed porous CoCrMo structures with different volume porosities of bcc unit cells with a length of 1 mm. The building direction was parallel to the longitudinal axis of the as-built CoCrMo samples. The chemical composition of fully dense as-built CoCrMo alloy was Co (58 wt%), Cr (28 wt%), Mo (6 wt%), and Si (<1 wt%).

### 2.2. Heat Treatment Process

The heat-treated CoCrMo structures were prepared using a combination of solution heat treatment at 1100 °C for 1 h and subsequent aging treatment at 800 °C for 4 h of the as-built CoCrMo alloys.

### 2.3. Mechanical Test

The uniaxial compression tests of as-built and heat-treated CoCrMo alloys were carried out using an HT-2402 universal testing machine produced by the Hung Ta Company, Taichung, Taiwan, with a 50 kN load cell and a strain rate of 2.1 × 10^−3^ s^−1^ at room temperature. The samples used for mechanical tests with dimensions of 5 mm × 5 mm × 4 mm were cut from the CoCrMo alloys. The compression direction was parallel to the building direction.

### 2.4. Microstructure Characterization

The CoCrMo alloys were mechanically polished using silicon carbide sandpapers of 4000-grit and finally using 0.02 µm colloidal silica suspension. The samples were subsequently etched for microstructure characterization using optical microscope (OM, Nikon ECLIPSE LV150N, Minato ku, Japan) and scanning electron microscopy (SEM, JEOL 6700F, Akishima, Japan).

### 2.5. Density Measurement

The density of the foam is shown below,

(1) $$\rho_{f}=\frac{M_{porous}}{V_{porous}}$$

where *M_porous_* and *V_porous_* are the weight and volume of the porous sample.

Meanwhile, the density of solid structure ($\rho_{S}$) was measured using the Archimedes method as follows,

(2) $$\rho_{S}=\frac{\rho_{w}\times w_{a}}{w_{a}-w_{w}}$$

where $\rho_{w}$ is the density of water, $w_{a}$ and $w_{w}$ are the weights of the sample in air and in water, respectively.

The relative density of material ($\rho^{*}$) was determined as

(3) $$\rho^{*}=\frac{\rho_{measured}}{\rho_{theoretical}}$$

where $\rho_{measured}$ and $\rho_{theoretical}$ are the measured density, and the theoretical bulk density, $\rho_{theoretical}$, is 8.3 g/cm^3^.

### 2.6. Finite Element Simulation

A finite element method using the ANSYS Explicit Dynamics (2019R1) software was simulated to predict the compressive mechanical behavior of the as-built and heat-treated porous CoCrMo with various designed porosities in comparison with the analytical prediction and experimental data. The 3D models with a length of 5 mm, a width of 5 mm, and a height of 4 mm exported from the 3D builder CAD software (18.0.1931.0), in STL file formats of the 3D-printed models were used for simulation to satisfy the actual compression test. The top and bottom plates were set as rigid bodies, while the 3D models were set to be deformable. The friction coefficient was set to 0.2, and the self-contact of 3D models was set. The Cartesian and tetrahedral mesh was used with a minimum element size of 0.00011 m, and the total number of the elements and nodes was around 220,000 and 59,000, respectively. The bottom plate had no freedom of x, y, and z displacements, and any rotation was restricted. The top plate was set with a z displacement while x and y displacements were restrained. The elastic and plastic behaviors were simulated based on an isotropic elasticity model and a bilinear isotropic hardening model, respectively. The values of Young’s modulus, yield strength, and tangential modulus derived from the experimental data of the fully dense as-built and heat-treated CoCrMo alloys were input into the simulation analysis. A Poisson’s ratio of 0.3 was applied [7]. A maximum plastic strain of 0.2 was set as the failure criterion of materials. If the plastic deformation of the element exceeded 0.2, it was considered damaged and thus removed.

## 3. Results

### 3.1. Morphology of As-Built and Heat-Treated Porous CoCrMo Alloys

**Figure 2:**
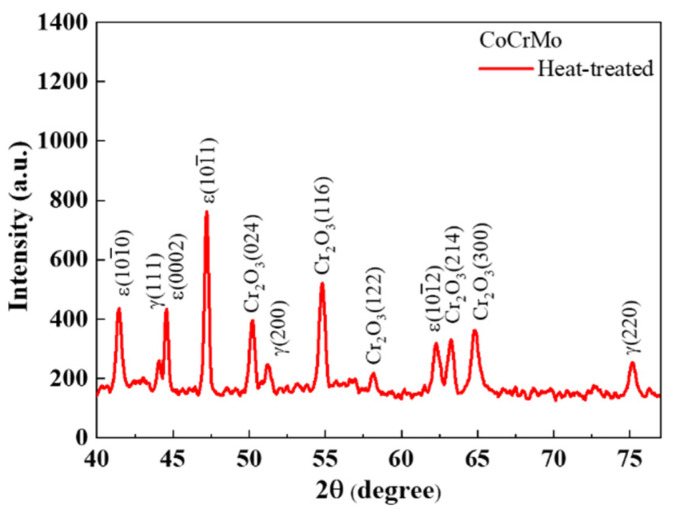
XRD profile on the surface of fully dense heat-treated CoCrMo alloy.

Figure 1f–o shows the cylinder shapes of the as-built and heat-treated CoCrMo alloys with different, designed volume porosities of 0, 20, 40, 60, and 80%. A discernable variation in surface morphology towards the rougher surfaces and the change in color in the sample surfaces was found using heat treatment. The heat-treatment-induced varying color from slight yellow to dark green was ascribed to the presence of a chromium surface oxide, which was demonstrated by an obvious appearance of Cr_2_O_3_ [48,49,50], as shown in the XRD patterns in Figure 2.

A martensitic transformation from an fcc to an hcp phase usually occurs in the CoCrMo alloys. Although the thermodynamically stable phase at room temperature is the hcp phase, the remaining fcc phase is mostly obtained in the CoCrMo alloys due to the sluggish transformation from the metastable fcc to the stable hcp under normal conditions [23,24]. The fcc to hcp transformation occurs more easily via rapid cooling, plastic deformation, and isothermal aging below the transformation temperature [23,24]. An examination of the degree of martensitic transformation via a heat treatment process was determined using XRD profiles in Figure 2. The as-built CoCrMo exhibited an obvious fcc phase with a lattice constant of 3.568 Å, while the heat-treated CoCrMo revealed the coexistence of residual fcc and hcp phases. The volume fractions of the hcp and fcc phases can be calculated as follows [25,51,52].

(4) $$f_{hcp}=\frac{I{\left(10\bar{1}1\right)}_{hcp}}{I{\left(10\bar{1}1\right)}_{hcp}+1.5I{\left(200\right)}_{fcc}}$$

(5) $$f_{fcc}=1-f_{hcp}$$

where $I{\left(10\bar{1}1\right)}_{hcp}$ and $I{\left(200\right)}_{fcc}$ are the integrated intensities of the ${\left(10\bar{1}1\right)}_{hcp}$ and ${\left(200\right)}_{fcc}$ diffraction peaks for the hcp and fcc phases, respectively.

The calculated volume fraction of the fcc and hcp phases in the fully dense heat-treated CoCrMo alloy were 64 and 36%, respectively.

### 3.2. Relative Density of the Solid Structures in Both Conditions

**Figure 3:**
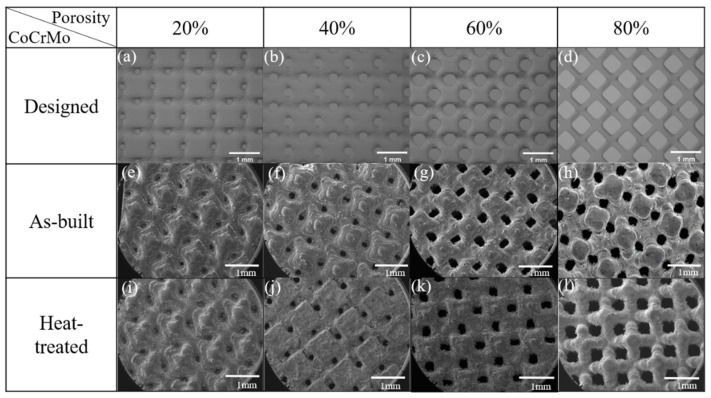
Top-view surface in (**a**–**d**) CAD-designed models, (**e**–**h**) as-built, and (**i**–**l**) heat-treated CoCrMo alloys with different, designed volume porosities.

Figure 3 shows the top-view surface in the as-built and heat-treated porous CoCrMo structures with different, designed volume porosities. The melt pool formed in different printing layers during SLM laser scanning was observed on the surface of fabricated samples, which was different from the smooth-designed model. The melt pool boundary became blurred, and the oxide layer, mainly composed of chromium oxide on the surface, made the surface rougher after heat treatment. In addition, there were also unmelted powder particles and spatter attached to the surface. When the melt pool was formed using a laser source, the excess heat energy would melt the nearby powders so they would adhere to the surface [39]. The distribution of pores was quite homogeneous in both conditions. Although the pore size on the surface of fabricated samples with a designed volume porosity of 20% was similar to that of the designed model, the pores were not as completely hollow in the vertical direction as those designed with the 3D model. The pores were obvious on the surface of fabricated samples with the designed volume porosity above 40%, and their pore sizes were evidently smaller than those of the designed model.

**Figure 4:**
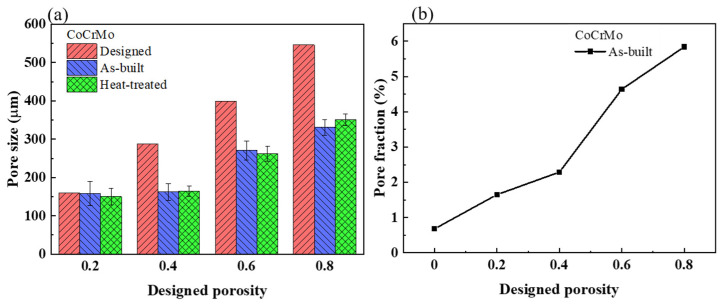
(**a**) Pore size in the designed, as-built, and heat-treated CoCrMo alloys with different, designed volume porosities. (**b**) Pore fraction in the as-built CoCrMo alloys with different, designed volume porosities.

Figure 4a describes the pore size in the designed, as-built, and heat-treated CoCrMo alloys. There was a negligible discrepancy in pore size between the as-built and heat-treated CoCrMo alloys. The difference in pore size between the fabricated and designed alloys increased with increasing designed volume porosity. As shown in the as-built CoCrMo alloys in Figure 4b, the pore fraction significantly increased with the increasing designed volume porosity.

**Figure 5:**
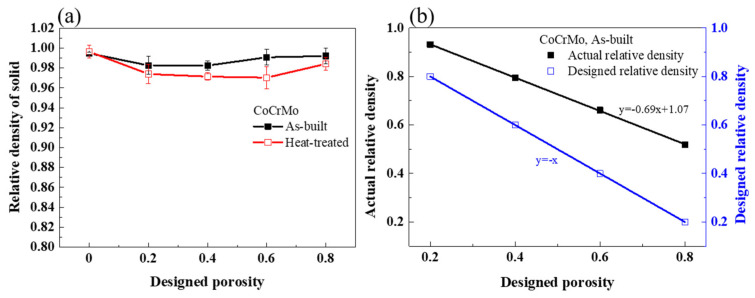
(**a**) The relative density of the solid in the as-built and heat-treated CoCrMo with various designed volume porosities. (**b**) The actual relative density and designed relative density in the as-built CoCrMo with various designed volume porosities.

Figure 5a describes the relative densities of the solids in the as-built and heat-treated CoCrMo alloys determined using Archimedes’ method to examine the printing quality of the SLM process. The relative densities of the fully dense as-built and heat-treated samples were 99.5 and 99.6%, respectively. The relative densities of the heat-treated porous CoCrMo alloys altered from 97 to 98.4%, which were slightly lower than the variation from 98.3 to 99.2% of the as-built porous samples. Such high relative densities suggested a negligible existence of internal void defects on the bulk struts during the fabrication process. The relative density of the solid was reduced slightly with an increasing designed volume porosity within the range of 20–40%; however, the effect of the designed volume porosity on the relative density of the solid was generally trivial. The lower relative densities of the solid seen at the designed volume porosity of 20–40% implied that a small number of voids was confined in the solid strut.

The actual relative densities in the as-built porous CoCrMo were determined using Equation (3) and compared with the designed relative densities in Figure 5b. The actual relative densities in the as-built CoCrMo alloys were all higher than the designed relative densities, in accordance with previous work [53]. Moreover, the difference between the actual and designed relative densities became larger with increasing designed volume porosity. It was possibly related to a thinner strut and lower thermal conductivity, which may enlarge the melt pool and increase the width of the strut compared to the designed model. Table 1 lists the parameters of the as-built porous CoCrMo alloys.

### 3.3. Compressive Deformation in the As-Built and Heat-Treated Porous CoCrMo Structures

**Figure 6:**
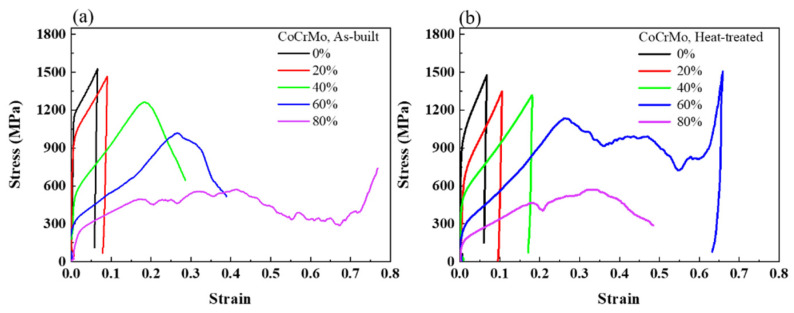
Compressive stress-strain curves of the (**a**) as-built and (**b**) heat-treated CoCrMo alloys with different, designed volume porosities.

Figure 6 presents the macroscopic stress-strain curves of uniaxial compression tests in both conditions. The three distinct deformation stages of linear elasticity, plateau, and densification were seen at the designed volume porosity above 40% in both conditions, which was similar to the typical deformation of porous structures proposed by Gibson–Ashby [54]. Young’s modulus was extracted in the initial stage of elastic deformation. The plastic deformation started to yield in the plateau region of the local collapse of pores, at which the strain significantly increased with a negligible variation in the stress. The plateau region was followed by a sharp increase in stress at the onset of the densification regime. The densification stages started at large strains of 0.65 and 0.74 in the heat-treated sample with a designed volume porosity of 60% and in the as-built CoCrMo with a designed volume porosity of 80%. In the present study, the SLM-built CoCrMo alloys with an actual porosity above 34% disclosed porous structures. Meanwhile, no obvious plateau and densification regions were obtained at a designed porosity below 40%, which is analogous to the deformation behavior of metallic solids. Such a drastic decline in stress after the elastic regime derived from the failure of struts in the porous structures owning high designed relative density.

**Figure 7:**
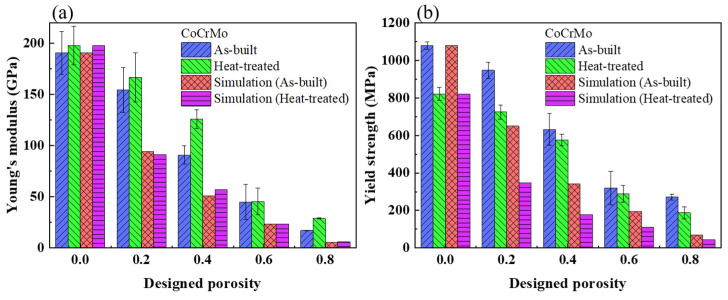
(**a**) Young’s modulus and (**b**) yield strength in the as-built and heat-treated CoCrMo alloys determined from the experiment and simulation with different, designed volume porosities.

Figure 7 shows the experimental and simulated values of Young’s modulus and the compressive yield strength in both conditions with respect to the designed volume porosity. The yield strength drastically decreased from 1079 to 822 MPa, while there was a slight increase in Young’s modulus from 191 to 198 GPa in the fully dense CoCrMo after heat treatment. The experimental values of Young’s modulus and the yield strength were generally higher than the simulated values in both conditions and may be due to a higher actual relative density rather than the designed relative density. Increasing the designed volume porosity significantly decreased Young’s modulus and the yield strength in both conditions. Furthermore, heat treatment resulted in a noticeable decrease in yield strengths but a slight increase in Young’s moduli for all different, designed volume porosities of CoCrMo alloys. Such a remarkable discrepancy in yield strength between the as-built and heat-treated conditions became smaller with increasing designed volume porosity. Among the investigated CoCrMo alloys, the as-built and heat-treated CoCrMo porous structures with the designed volume porosity of 80% had Young’s moduli of 17 and 29 GPa, respectively, while they possessed compressive yield strengths of 271 and 187 MPa, respectively. Compared to Young’s modulus of 3–30 GPa and a yield strength of 193 MPa in the human cortical bone [18,55], the as-built and heat-treated CoCrMo porous structures with a designed volume porosity of 80% were the most appropriate implants for potential biomedical applications due to a very close match in their mechanical responses. In addition, heat treatment was more conducive to tailoring the mechanical performance of SLM-built porous structures, which was more similar to that of human cortical bone. The mechanical properties of CoCrMo structures could be effectively tuned using an adjustable, designed volume porosity fabricated via SLM.

### 3.4. The Compressive Mechanical Properties Using the Gibson–Ashby Model and Finite Element Simulation

An analytical model proposed by Gibson–Ashby enables the effective prediction of the adjustable porosity for isotropic materials [54]. The compressive mechanical responses of porous CoCrMo structures were fitted using the Gibson–Ashby model to evaluate the degree of matching among the analytical, simulated, and experimental results in establishing a more suitable design of porous structures for potentially promising implants in biomedical applications. The compressive deformation of porous CoCrMo alloys was implemented using finite element simulation. The analytical prediction of an elastic modulus and yield strength was presented with the Gibson–Ashby model as follows [54].

(6) $$\frac{E}{E_{S}}=C_{1}{\left(\frac{\rho }{\rho_{S}}\right)}^{n}$$

(7) $$\frac{\sigma }{\sigma_{S}}=C_{5}{\left(\frac{\rho }{\rho_{S}}\right)}^{m}$$

where *E* and $E_{S}$ are the elastic modulus of cellular and solid materials, respectively, σ and $\sigma_{S}$ are the yield strength of cellular and solid materials, respectively, ρ and $\rho_{S}$ are the density of cellular and solid materials, respectively, *C*_1_ and *C*_5_ are constants, and *n* and *m* are exponential factors. The general values of *n* and *m* are 2 and 1.5, respectively. *C*_1_ and *C*_5_ are ideally 1; however, their variable values were experimentally determined from the best-fitting curve.

**Figure 8:**
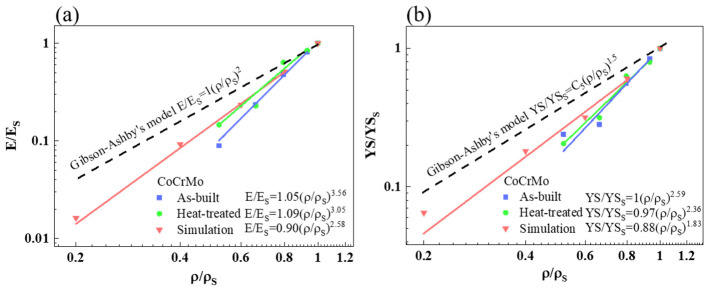
(**a**) The relative elastic modulus and (**b**) relative yield strength obtained from the analytical, simulated, and experimental results versus a logarithmic scale of relative density in the as-built and heat-treated CoCrMo alloys.

The relative elastic moduli $E/E_{S}$ and relative yield strengths $\sigma /\sigma_{S}$ obtained from the analytical, simulated, and experimental results versus a logarithmic scale of relative density were plotted in Figure 8. The fitted value of *C*_1_ from the simulated and experimental results was in the range of 0.9–1.09. The variation in *n* is mainly related to the predominant deformation mode of strut-based cellular structures, which can be determined based on the lattice structure of repeating unit cells. Based on the Maxwell number of the bcc unit cell [56,57], the compressive deformation mechanism of the porous CoCrMo alloys was ascribed to the bending-dominated deformation proposed using the Gibson–Ashby model in which the exponent *n* is 2. The fitting curves of relative elastic modulus in both simulated and experimental results revealed linear relation with respect to the relative density, following the power law relationship. In Figure 8a, the exponent *n* extracted from the fitting curves of the simulated and experimental results was larger than that from the analytical Gibson–Ashby model. The fitting curve of the simulation analysis deviated from that of the Gibson–Ashby model, and it has an exponent of 2.58, which was close to the exponential value obtained in the simulation analysis of other bcc strut-based structures [45]. The fitting curve of as-built and heat-treated samples was closer to that of the simulation with an exponent of 3.56 and 3.05, respectively, which was far outside the expected range. The data of the simulation analysis and experiment were all well-fitted.

In Figure 8b, the relative yield strength versus relative density also followed a linear trend, which was similar to the relative elastic modulus versus relative density. The fitting curve of the relative yield strength in both simulated and experimental results also deviated from that proposed using the Gibson–Ashby model. The data of simulation analysis and heat-treated samples were better fitted, while there was a deviation between the fitting curve and data in the as-built samples. The exponent *m* obtained from the simulated, as-built, and heat-treated results were 1.83, 2.59, and 2.36, respectively, which was relatively greater than the 1.5 derived from the Gibson–Ashby model. The exponential value of 1.83 derived from simulation analysis was close to that of 1.97 in other bcc strut-based structures [45]. In general, a closer agreement was attained in the simulation analysis of bcc strut-based structures between this study and another previous study [45]. However, there was not a close correlation between the predicted and experimental values in the present study.

### 3.5. Microstructural Characterization in Both Conditions

**Figure 9:**
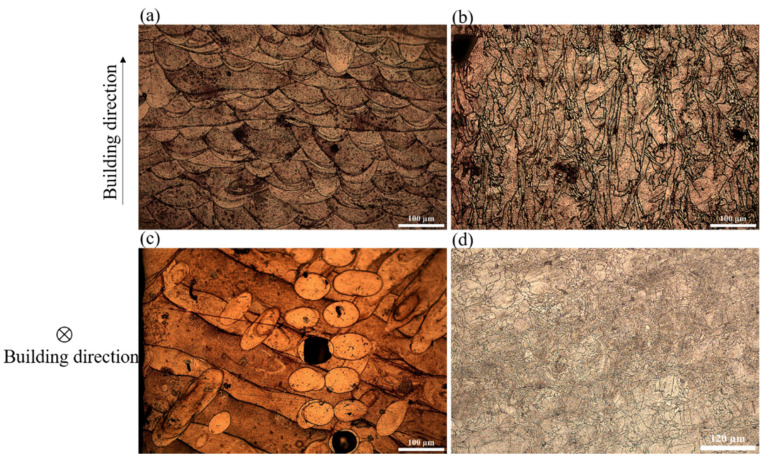
OM micrographs in the fully dense (**a**) as-built and (**b**) heat-treated CoCrMo alloys in the plane parallel to the building direction; (**c**) and (**d**) Those in the plane perpendicular to the building direction, respectively.

The microstructures significantly govern the mechanical performance of SLM-built alloys. A considerably decreased yield strength in the heat-treated CoCrMo alloys was ascribed to the microstructural change after heat treatment. Figure 9 depicts OM images of the morphologies in the fully dense as-built and heat-treated CoCrMo alloys. In Figure 9a,c, two typical morphologies of melt pools in half-cylinder and stripe-like shapes were obvious in the plane parallel and perpendicular to the building direction, respectively, which was similarly seen in other SLM-built alloys. In Figure 9b,d, there was a disappearance of melt pools and a presence of grain boundaries with different grain sizes after heat treatment. In Figure 9b, most of the large grains were elongated with their long axes parallel to the building direction, implying an incomplete recrystallization process after heat treatment. The average length and width of elongated grains were 120 and 44 μm, respectively. Fine grains were also observed, which were newly formed grains in the initial stage of recrystallization. In Figure 9d, the grains were more likely to be equiaxed grains with an average size of 11 μm.

**Figure 10:**
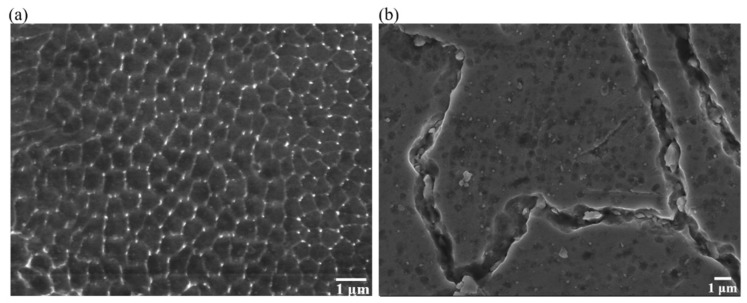
SEM images in the fully dense (**a**) as-built and (**b**) heat-treated CoCrMo alloys.

Figure 10 shows SEM images in the fully dense as-built and heat-treated CoCrMo alloys. A typical cell structure with an average size of 0.57 μm was obvious in the as-built CoCrMo, shown in Figure 10a. The energy-dispersive X-ray spectroscopy (EDS) analysis disclosed a different elemental distribution between the cell boundary and the cell. The cell boundary was found to be C-Mo rich, presumably ascribed to the M_23_C_6_ phase [29]. When the metal powder was heated and cooled rapidly, Mo with a high melting point was discharged to the cell boundary, and CoCr remained inside the cell [58]. In Figure 10b, the formation of precipitates was visible at both the grain boundaries and within the grains after heat treatment. The precipitates at the grain boundaries seemed to be slightly elongated along the grain boundaries, and their sizes were much greater than those inside the grains.

### 3.6. Strengthening Mechanisms

The mechanical properties of CoCrMo alloys were significantly altered in the as-built and heat-treated conditions, ascribed to their microstructural changes in governing the role of distinct strengthening behaviors, such as grain boundary strengthening, dislocation strengthening, and Orowan strengthening. The contribution of each strengthening mechanism to the calculated yield strength of CoCrMo alloys in both conditions was estimated.

The grain boundary strengthening is shown below [24,59].

(8) $$\Delta \sigma_{GB}=kd^{-1/2}$$

where *k* is the Hall–Petch constant, and it has different values for the fcc and hcp phases. *K* is 400 [6] or 243.9 MPa μm^−1/2^ [60] for the fcc or hcp phase, respectively. *d* is the average grain or cell size determined from SEM analysis.

The dislocation strengthening was described as follows [24].

(9) $$\Delta \sigma_{dis}=\alpha MGb\rho_{dis}{}^{1/2}$$

where α is a dimensionless constant, *M* is the Taylor factor, *G* is the shear modulus, *b* is the Burgers vector, and $\rho_{dis}$ is the dislocation density. α is 0.24 [61] or 0.1 [62] for the fcc or hcp phase, respectively. *G* is 78.4 [63] or 82.2 GPa [64] for the fcc or hcp phase, respectively. *b* is 0.1463 nm for both the fcc and hcp phases [14]. $\rho_{dis}$ was obtained from CMWP fitting.

The Orowan mechanism was expressed as [65].

(10) $$\Delta \sigma_{Orowan}=\frac{2Gb}{d_{f}}{\left(\frac{6V_{f}}{\pi }\right)}^{1/3}$$

where $d_{f}$ is the average diameter of precipitates and $V_{f}$ is the volume fraction of precipitates. $d_{f}$ and $V_{f}$ were determined from SEM analysis.

The calculated yield strength for the fcc or hcp phase was presented as

(11) $$\sigma_{i}=M\tau_{CRSS}+\Delta \sigma_{GB}+\Delta \sigma_{dis}+\Delta \sigma_{Orowan}$$

where *i* is the fcc or hcp phase, *M* is the Taylor factor, and $\tau_{CRSS}$ is the critical resolved shear stress [66]. *M* is 2.57 [66] or 3.06 [25] for the fcc or hcp phase, respectively. $\tau_{CRSS}$ is 54 [67] or 184 MPa [62] for the fcc or hcp phase, respectively.

There was a negligible contribution of precipitation hardening, and only the fcc phase existed in the as-built CoCrMo alloys. Meanwhile, since there existed two phases of fcc and hcp after heat treatment, the calculated yield strength of heat-treated CoCrMo alloys could be estimated with the rule of mixture as shown below [24].

(12) $$\sigma_{y}=f_{hcp}\sigma_{hcp}+\left(1-f_{hcp}\right)\sigma_{fcc}$$

where $f_{hcp}$ is the volume fraction of hcp grains, and $\sigma_{hcp}$ and $\sigma_{fcc}$ are the strengths of hcp and fcc grains, respectively.

**Figure 11:**
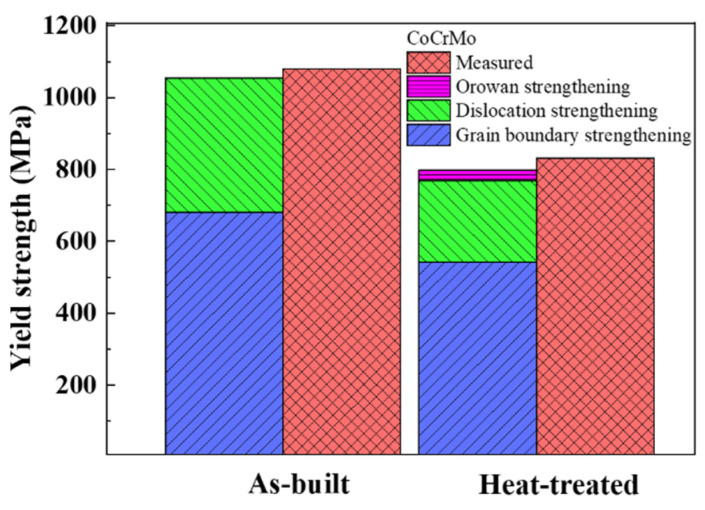
The measured and calculated yield strengths consisting of the grain boundary strengthening, dislocation strengthening, and Orowan strengthening in the fully dense as-built and heat-treated CoCrMo alloys.

Figure 11 describes the contributing strength values and calculated yield strengths compared with the measured yield strengths in the fully dense as-built and heat-treated CoCrMo alloys. The calculated yield strengths in the as-built and heat-treated CoCrMo alloys were 1055 and 790 MPa, respectively, which were in very good accordance with the measured yield strengths of 1079 and 822 MPa, respectively. Both the strength values of grain boundary strengthening and dislocation strengthening were reduced after heat treatment. The disappearance of cellular structure, the increase in grain size, and the decrease in dislocation density were mainly responsible for the decreased yield strength after heat treatment.

## 4. Discussion

The Gibson–Ashby model is one of the most popular theoretical predictions to evaluate the correlation between porous structures and their mechanical performance. Clarifying reasonable factors governing the difference between the predicted and experimental results is necessary to establish a better design for SLM-built porous CoCrMo structures. The predicted values from simulation and analytical analysis underestimated the elastic modulus and yield strength of the CoCrMo alloys as compared to the experimental values. Higher exponential values derived from the experimental data of elastic modulus and yield strength were presumably attributed to the structural characteristics of the bcc strut-based structures. The strut was not parallel to the compression direction, which was different from the Gibson–Ashby model. Thus, the resistance to load deformation is weak and results in larger exponential factors of elastic modulus and yield strength [68]. Another possible reason is the presence of defects during the SLM process, causing the discrepancy between the designed and actual relative density [69]. Due to the increased surface area of the strut, the printing process causes a partial melting of the loose powder beneath it and bonds it to the strut surfaces, resulting in the increased weight and higher actual relative density of the specimen but a negligible contribution to the mechanical strength [45,69]. In addition, the corrugation and increased surface roughness of the strut caused by partially melted powder possibly induce stress concentration and thus lead to a lower elastic modulus as well as a lower yield strength of the SLM-built alloys [69]. As the relative density decreases, the thinner the strut, the slower the cooling rate, and the coarser the microstructure, which acts as another reasonable factor in reducing the yield strength besides the density effect [70]. Such a lower yield strength results in an increase in the exponential factor of the experimental data. Although the elastic modulus and yield strength were not well fitted using the Gibson–Ashby model, the predicted values could still be referred to for future designs of SLM-built porous CoCrMo alloys with adjustable mechanical properties.

## 5. Conclusions

The role of different, designed volume porosities and heat treatment processes on the mechanical properties of SLM-built CoCrMo alloys were investigated. The SLM-built CoCrMo with an actual porosity above 34% exhibited porous structures. An optimal actual porosity of 48% resulted in appropriate mechanical responses of the SLM-built CoCrMo structures compared to those of the human bone. Furthermore, the heat treatment process was found to be more beneficial in tailoring Young’s modulus and the yield strength of the SLM-built CoCrMo alloys with a minimal stress shielding effect. Possible explanations for the underestimated exponential factors from simulation and analytical analysis compared to the experimental values were reported. Our findings suggest the optimal design of bcc lattice-structure-based CoCrMo alloys for a closer match in mechanical properties between the porous SLM-built CoCrMo implants and bone tissue for potential biomedical applications.

## Figures and Tables

**Table 1:** Parameters of the as-built porous CoCrMo alloys.

Designed Volume Porosity (%)	Designed Relative Density (%)	Actual Relative Density (%)	Relative Density of Solid (%)
20	80	93.3	98.3
40	60	79.5	98.2
60	40	66.2	99.1
80	20	52.1	99.2

## Data Availability

The data presented in this study are available on request from the corresponding author.

## Nomenclature

bcc	Body-centered cubic
SLM	Selective laser melting
hcp	Hexagonal closest packed
Ta	Tantalum
Ti	Titanium
Co	Cobalt
CoCrMo	Cobalt-chromium-molybdenum
3D	Three-dimensional
CAD ITRI	Computer aided design Industrial Technology Research Institute
OM	Optical microscope
SEM	Scanning electron microscopy
$\rho_{f}$	Density of foam
$\rho_{S}$	Density of solid structure
*M_porous_*	Weight of the porous sample
*V_porous_*	Volume of the porous sample
$\rho_{S}$	Density of solid structure
$\rho_{w}$	Density of water
$w_{a}$	Weight of the sample in air
$w_{w}$	Weight of the sample in water
$\rho^{*}$	Relative density of material
$\rho_{measured}$	Measured density
$\rho_{theoretical}$	Theoretical bulk density
$f_{hcp}$	Volume fraction of hcp
$f_{fcc}$	Volume fraction of fcc
$I{\left(10\bar{1}1\right)}_{hcp}$	Integrated intensity of the ${\left(10\bar{1}1\right)}_{hcp}$ peaks for the hcp
$I{\left(200\right)}_{fcc}$	Integrated intensity of the ${\left(200\right)}_{fcc}$ peaks for the fcc
*E/E_S_*	Relative elastic modulus
$\sigma /\sigma_{S}$	Relative yield strength
*E*	Elastic modulus of cellular material
*E*_S_	Elastic modulus of solid material
*σ*	Yield strength of cellular material
*σ_S_*	Yield strength of solid material
*ρ*	Density of cellular material
*ρ_S_*	Density of solid material
*C*_1_	Constant
*C*_5_	Constant
*m*	Exponential factor
*n*	Exponential factor
EDS	Energy-dispersive X-ray spectroscopy
$\Delta \sigma_{GB}$	Grain boundary strengthening
*k*	Hall-Petch constant
*d*	Average grain or cell size
$\Delta \sigma_{dis}$	Dislocation strengthening
*α*	Dimensionless constant
*M*	Taylor factor
*G*	Shear modulus
*b*	Burgers vector
$\rho_{dis}$	Dislocation density
$\Delta \sigma_{Orowan}$	Orowan strengthening
$d_{f}$	Average diameter of precipitates
$V_{f}$	Volume fraction of precipitates
*i*	fcc or hcp phase
$\tau_{CRSS}$	Critical resolved shear stress
$\sigma_{hcp}$	Strength of hcp
$\sigma_{fcc}$	Strength of fcc
